# MRI-Related Claustrophobia: Patient-Reported Experience and Associated Factors in a Makkah Region Cohort

**DOI:** 10.3390/tomography12060077

**Published:** 2026-05-25

**Authors:** Shrooq T. Aldahery, Lubna A. Bushara, Rana A. Alasami, Mona H. Alqurashi, Rahaf O. Alqurayqiri, Sahar E. Behilak, Faten S. Kandil, Khalid M. Alshamrani, Walaa M. Alsharif, Awadia Gareeballah, Fahad H. Alhazmi, Mohammed S. Almatrafi

**Affiliations:** 1 Department of Applied Radiologic Technology, College of Applied Medical Sciences, University of Jeddah, P.O. Box 80327, Jeddah 21589, Saudi Arabia; labushara@uj.edu.sa (L.A.B.); ralasami.stu@uj.edu.sa (R.A.A.); malqurashi00812019.stu@uj.edu.sa (M.H.A.); rahmad0017.stu@uj.edu.sa (R.O.A.); 2 Department of Nursing, College of Applied Medical Sciences, University of Jeddah, P.O. Box 80327, Jeddah 21589, Saudi Arabia; sebehilak@uj.edu.sa (S.E.B.); fskandil@uj.edu.sa (F.S.K.); 3 College of Applied Medical Sciences, King Saud bin Abdulaziz University for Health Sciences, Jeddah P.O. Box 22384, Saudi Arabia; alshamranik@ksau-hs.edu.sa; 4 King Abdullah International Medical Research Center, Jeddah P.O. Box 22384, Saudi Arabia; 5 Ministry of the National Guard-Health Affairs, Jeddah P.O. Box 22384, Saudi Arabia; 6 Department of Diagnostic Radiology, College of Applied Medical Sciences, Taibah University, P.O. Box 30002, Madinah 42353, Saudi Arabia; wsheref@taibahu.edu.sa (W.M.A.); agsali@taibahu.edu.sa (A.G.); fhdhazmi@taibahu.edu.sa (F.H.A.); 7 Medical Centre, Taibah University, P.O. Box 30002, Madinah 42353, Saudi Arabia; msmatrafi@taibahu.edu.sa

**Keywords:** MRI claustrophobia, patient anxiety, magnetic resonance imaging, patient experience, demographic predictors, patient education, MRI technologist communication, coping strategies, Saudi Arabia

## Abstract

**Purpose**: This study aimed to assess MRI-related claustrophobia severity and patient-reported experiences among Saudi patients to examine their associations with selected demographic variables. **Methodology**: A cross-sectional study was conducted using a structured questionnaire administered to 200 Saudi patients who had previously undergone MRI examinations. The questionnaire comprised five sections covering demographic data, phobia severity and patient-reported experiences before, during and after MRI examinations. Statistical analysis was performed using SPSS statistical package (IBM SPSS Statistics version 26, IBM Corp., Armonk, NY, USA), applying chi-square tests to examine associations between demographic variables and questionnaire responses. **Results**: A significant majority of participants, 76.5%, reported a positive MRI experience, whereas only 6.5% reported a negative experience. Shortness of breath during the MRI examination was the most frequently reported source of discomfort (75%). Significant associations were identified between demographic characteristics and phobia severity. Age and gender were significantly correlated with sudden fear responses, while educational level was strongly associated with receiving adequate pre-scan information and overall examination experience. **Conclusions**: Despite the high percentage of positive experiences, a notable proportion of participants reported anxiety-related distress during MRI examinations. The observed associations between demographic variables and claustrophobia-related responses suggest the potential value of patient-centred approaches, particularly improved pre-scan education, to improve the MRI-related patient experience and reduce anxiety-related distress.

## Simple Summary

Magnetic resonance imaging (MRI) is an important diagnostic tool, yet some patients experience claustrophobia and anxiety during MRI examinations. This study investigated the severity, patient-reported experience and demographic correlates of MRI-related claustrophobia among 200 patients in Saudi Arabia who had previously undergone MRI scans. Participants completed a structured questionnaire covering demographic characteristics, phobia severity, and experiences before, during and after MRI. Although most participants reported an overall positive MRI experience, a notable proportion described anxiety-related symptoms, with shortness of breath a common complaint. Age showed the strongest associations with claustrophobia severity, followed by gender, weight and educational level. In addition, better pre-scan information and effective communication with MRI technologists were associated with more favourable patient-reported experiences. These findings highlight the importance of patient education and supportive communication in improving MRI-related patient experiences.

## 1. Introduction

Claustrophobia is an anxiety disorder that is defined as a specific fear of enclosed spaces, such as fear of restricted rooms, underground tunnels, vaults, elevators, magnetic resonance imaging (MRI) units, aeroplanes or crowded places [1]. Approximately 12.5% of people experience claustrophobia. A typical person with a particular phobia, such as claustrophobia, fears three objects or situations. Of those who have a specific phobia, 75% dread more than one object or situation [2]. People with specific phobias generally avoid situations that trigger their fear. The fear can be expressed in physical symptoms such as difficulty breathing, pulsing, sweating, tachycardia, dry mouth or chest pain, or in emotional symptoms such as overpowering anxiety, fear of losing control or an intense need to leave the situation. The fear is understood as illogical, yet it presents with an inability to overcome it. It may sometimes develop because of traumatic incidents experienced during childhood [3]. Avoidance coping is the preferred coping method for those who struggle with anxiety and phobias; this is especially true of claustrophobia, which is avoidance coping. This is because it acts as a transient barrier to restrain the anticipated panic and undesirable feelings connected to enclosed areas [4]. However, any coping technique that relies on avoidance can only make the phobia worse. This is because people struggle to learn how to deal with triggering situations and anticipate panic, both of which can amplify anxiety and fear [5].

MRI is increasingly being used as a non-invasive diagnostic technique for various medical conditions. The patient must typically be positioned in the middle of a large magnet bore in a constrained space to optimize image quality [6]. The patient must be inserted headfirst or feetfirst into the MRI bore and must stay inside for the duration of the scan, typically ranging from 15 to 45 min, depending on the examination protocol [7]. In addition, depending on the region being examined, specific coils are used to maintain image quality [8]. According to previous studies, 15% of patients experience acute claustrophobic symptoms when inside the MRI bore [9,10]. These reactions may cause the scan to end prematurely or to be cancelled and rescheduled, which may create an additional workflow burden and delay evaluation or degrade the quality of the obtained images [11]. During MRI examinations, high levels of anxiety can lead to increased patient movement; such movement can result in motion artefacts, which can negatively impact diagnostic accuracy [12]. Preserving image quality is essential for accurate diagnosis, and recent advances in image processing and explainable artificial intelligence (XAI) have further highlighted the importance of minimizing factors that degrade diagnostic image quality, including motion-related artefacts during MRI examinations [13].

It has also been reported that two million scans worldwide cannot be completed each year, either because they are stopped prematurely or because the patient refuses to be scanned due to claustrophobia [14]. Anxiety during an MRI scan may be caused by claustrophobia or other conditions, including apprehension about medical personnel, prospective diagnosis or ambient circumstances [15]. The capacity to effectively diagnose a patient is impacted by these missed scans and represents a considerable resource cost to health organisations [16]. As manufacturers became aware of rising expenses due to claustrophobia and anxiety-related events, advancements in the design of MRI scanners were made to make them more patient-friendly. Recent advancements in MRI design include shorter and broader bores, open scanners and lower noise levels [17].

Despite technological developments, MRI examinations are still intimidating and have not completely reduced claustrophobic reactions. Many patients who attend MRI departments might be well educated about the examination. Others, however, may feel nervous or uncomfortable with the thought of being on their back for an amount of time during the examination [18]. In addition, the attitudes and behaviours of healthcare providers have a significant impact on patients’ experiences. MRI technologists can improve the experience by explaining the examination, reassuring patients and establishing a connection with them. On the other hand, they can adversely affect the patient’s experience by being brusque or inattentive because patients who are being scanned depend on them to provide the required information about the examination [19]. Therefore, a lack of information from the technologist can cause anxiety in the patients during examination [20]. Al-Shemmari et al. (2022) [21] demonstrated the effectiveness of several strategies for assisting claustrophobic patients during MRI examinations, thereby improving patient experience and increasing scan completion rates. These strategies include providing patients with information about the scan, using wide-bore or open MRI scanners, allowing the presence of a family member and, more recently, using virtual reality tools [21]. In addition, patient knowledge about MRI scanners is imperative to overcome the challenge of experiencing anxiety during the examination. Knowledge can be enhanced by providing educational materials such as posters, brochures or videos demonstrating machine configuration, shape and the nature of the exam [22]. Despite the growing use of MRI and the recognized impact of claustrophobia on patient experience, there remains limited region-specific evidence examining patient-reported claustrophobia and its associated factors within the Saudi population. In addition, few studies have comprehensively explored how demographic characteristics and modifiable factors, such as patient education and communication with MRI technologists, influence patient experience. This study aimed to assess the severity and patient-reported experience of MRI-related claustrophobia and anxiety in the Makkah region cohort and to examine their associations with selected demographic characteristics. The study further sought to explore the relationship between patient preparation, communication, and coping strategies with the overall MRI experience. It was hypothesized that patient-related factors, including demographic characteristics, level of pre-scan information, and interaction with MRI technologists, would significantly influence the severity of claustrophobia and overall patient experience during MRI examinations.

## 2. Methods

### 2.1. Study Design and Setting

A quantitative descriptive design utilising a cross-sectional survey approach was employed to assess the attitudes and awareness of claustrophobia during MRI procedures among the patient population in the Makkah region of Saudi Arabia. Data collection took place from 20 October to 19 December 2025. The study utilised a multi-site approach so that the survey was distributed to patients attending multiple radiology departments and imaging centres across the Makkah region. The study included both public hospitals and private clinics where MRI examinations are routinely performed. Participants were recruited using a convenience sampling approach from patients attending radiology departments and imaging centers in the Makkah region, including both public hospitals and private clinics. A total of 437 participants responded to the questionnaire; however, 237 were excluded because they did not meet the inclusion criteria of having undergone an MRI scan. This resulted in a final analytical sample of 200 participants. Participants who had never undergone an MRI examination were explicitly excluded from the study. Participants were eligible for inclusion if they had previously undergone at least one MRI examination. Individuals who had never undergone an MRI examination were excluded from the study. Only fully completed questionnaires were included in the final analysis. The MRI examinations were performed using different MRI systems. A formal a priori sample size calculation was not performed because the study employed a convenience sampling approach and was exploratory in nature. However, the final sample was considered adequate for detecting moderate associations between categorical variables using Chi-square analysis at a significance level of *p* < 0.05 with acceptable statistical power. In addition, the sample size satisfied the expected cell count assumptions for Chi-square testing and was comparable to sample sizes reported in similar MRI anxiety and claustrophobia studies [13]. MRI examinations were conducted according to routine clinical protocols at each participating center, depending on the anatomical region and clinical indication. As this study focused on patient-reported experiences rather than imaging performance or diagnostic outcomes, detailed imaging acquisition parameters (e.g., TR, TE, flip angle) were not collected. Detailed scanner specifications (e.g., field strength such as 1.5 T or 3 T) were not collected, as the study focused on patient-reported experiences rather than technical scanner parameters. However, participants were asked to report whether the examination was performed in an open or closed MRI system. Variations in scanner design and field strength may influence patient comfort and claustrophobia levels and should be considered when interpreting the findings.

### 2.2. Ethical Approval

Ethical approval was sought and received from the Bioethics Committee for Scientific and Medical Research at the University of Jeddah (Reference Number: HAP-02-J-094—Application number: UJ-REC-367). The approval process involved a multidisciplinary review to ensure that the research goals were clearly understood and that the dignity, rights, safety and well-being of the research participants were protected throughout the study.

### 2.3. Study Tool and Questionnaire Validation

Data was collected using a structured self-administered questionnaire consisting of demographic and MRI experience items developed based on a review of relevant literature [18,23]. The questionnaire comprised 36 questions organised into five distinct sections. The first section involved the demographic profile of the participants, which included their gender, age, weight, education level, nationality, etc. The second section involved the phobia severity measure, which focused on determining the severity scale for specific phobias. The phobia severity section consisted of an Arabic-translated version of the DSM-5 Severity Measure for Specific Phobia—Adult developed by the American Psychiatric Association. The scale included 10 items assessing emotional, cognitive, behavioral, and physical manifestations of claustrophobia-related symptoms during MRI examinations. The third part involved questions about patient experiences before the examination. The fourth section explored the patient experience during the examination. The fifth section collected information about the patient experience after the examination. The question regarding the most uncomfortable feeling during the MRI examination was presented as a multiple-choice item with predefined response options (extreme cold, shortness of breath, feeling confined in the examination room for a prolonged period, remaining still for a long time or other). The questionnaire used for data collection is provided in Supplementary File S1. Each response option was coded as a categorical variable to allow statistical comparisons between demographic characteristics. A pilot test was conducted with a small group of patients to confirm clarity, reliability and feasibility prior to full distribution. Based on participants’ feedback, adjustments were implemented, including rephrasing complex medical terms, optimising question order to enhance the flow and prevent confusion among the patients and removing redundant questions. The revised questionnaire was subsequently reviewed and validated by a panel of experts in radiology, psychology and medical research to ensure strong content validity.

### 2.4. Data Collection Technique

The questionnaire was digitised using Google Forms to facilitate distribution to participants via a variety of online portals, including social media applications, email and WhatsApp, as part of the recruitment process. These platforms were used only to distribute the survey link, while responses were submitted directly through the Google Forms interface. The form was configured not to collect email addresses or any identifying personal information, ensuring that responses remained anonymous. The questionnaire briefly explained the research goals and the identity of the researchers conducting the study. In addition, it confirmed that informed consent was obtained from each participant. Participants were explicitly informed that their participation was voluntary and that their information would remain completely anonymous and confidential. All responses were automatically stored within the secure Google Forms database and were accessible only to the research team. To minimise missing data, all questionnaire items were configured as required fields in Google Forms, ensuring that participants could not submit the survey without completing all questions. Consequently, all submitted questionnaires were complete and included in the final analysis.

### 2.5. Statistical Analysis

All collected data were meticulously analysed using the SPSS statistical package (IBM SPSS Statistics version 26, IBM Corp., Armonk, NY, USA). To evaluate the overall level of claustrophobia severity, a composite score was calculated for each participant based on responses to the 10-item Arabic-translated DSM-5 Severity Measure for Specific Phobia—Adult included in the questionnaire. Each item assessed a specific manifestation of claustrophobia, including emotional responses (e.g., sudden fear or anxiety), cognitive responses (e.g., thoughts of injury or negative outcomes), behavioural responses (e.g., avoidance or preparation behaviours) and physical symptoms (e.g., racing heart or muscle tension). Responses were coded on a five-point Likert scale ranging from 0 = never to 4 = all the time and summed to generate a total severity score for each participant. For item-level association analyses, responses to individual questionnaire items were treated as categorical variables and analyzed using Chi-square tests to examine associations with demographic characteristics and patient experience variables. The possible total score ranged from 0 to 40, with higher scores indicating greater severity of claustrophobic symptoms during MRI examinations. The chi-square test was used to compare demographic groups in terms of the distribution of responses for each question, thereby examining the associations between demographic variables and participant responses, as it is an appropriate method for analyzing associations between categorical variables derived from questionnaire responses. Internal consistency of the 10-item Arabic-translated phobia severity scale was evaluated using Cronbach’s alpha. All statistical tests were conducted at a significance level of *p* < 0.05.

## 3. Results

The final sample comprised 200 participants who had previously undergone MRI examinations, of whom 18.5% were male, and 81.5% were female (Table 1). Most participants, 51.5%, were young adults aged 18–29 years, while 32.5% were older than 40 years. Most respondents had a bachelor’s degree (68.5%), and the majority were Saudi nationals (96%). Regarding general claustrophobia-related symptoms, 27% of participants reported experiencing shortness of breath in narrow spaces, while 29.5% reported possible symptoms. In addition, 34% reported hearing about negative MRI experiences from relatives or friends, which might have influenced patient expectations prior to the examination.

**Figure 1:**
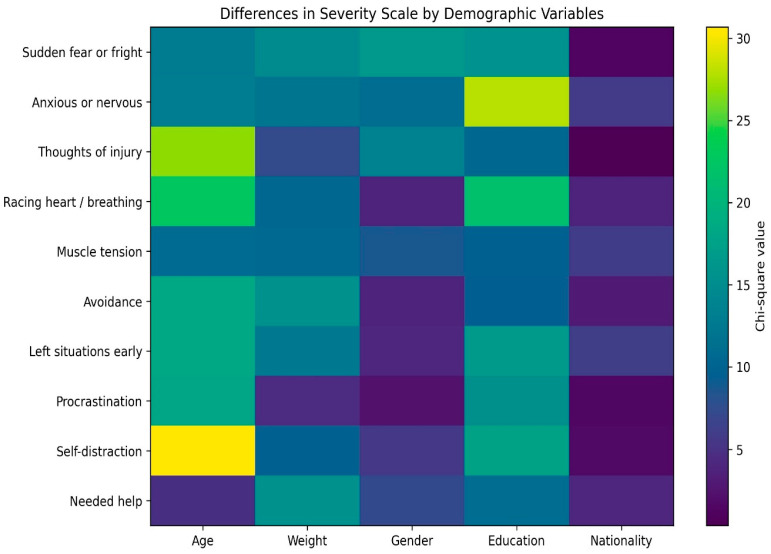
Differences in the phobia severity scale according to demographic variables.

Figure 1 illustrates the phobia severity score, which ranged from 0 to 18, with higher scores indicating greater severity of claustrophobic symptoms during MRI examinations. The 10-item Arabic-translated phobia severity scale demonstrated excellent internal consistency, with Cronbach’s alpha = 0.909. The chi-square analysis explored the relationships between the demographic variables and the items on the phobia severity scale. Age demonstrated several statistically significant associations with phobia severity items (*p* < 0.05), including sudden fear, thoughts of injury, racing heart, breathing difficulties, and avoidance behaviors. One of the highest Chi-square values observed in the analysis was between age and the coping strategy of self-distraction (χ^2^ = 30.69, *p* < 0.01), suggesting possible differences in coping mechanisms across age groups. Weight and gender also demonstrated several significant associations, including MRI machine type (χ^2^ = 6.95, *p* < 0.05) and use of anti-anxiety medication (χ^2^ = 8.81, *p* < 0.05). In addition, both variables were associated with several severity measures, particularly sudden fear, anxiety symptoms, and avoidance behaviors (*p* < 0.05). Educational level showed a strong association, particularly receiving sufficient pre-scan information (χ^2^ = 41.23, *p* < 0.01) and MRI machine type (χ^2^ = 21.23, *p* < 0.01). In contrast, nationality did not show statistically significant associations with the severity measures (*p* > 0.05), indicating that these responses were largely independent of participants’ national background.

Table 2 presents the differences in patient knowledge and MRI examination characteristics according to demographic variables. Significant associations were observed for several items (Table 2). Age showed a significant association with the use of anti-anxiety medication before the scan (χ^2^ = 11.72, *p* < 0.05). Weight was significantly associated with the type of MRI machine used for the examination (open vs. closed) (χ^2^ = 6.95, *p* < 0.05). Gender was also significantly associated with the use of anti-anxiety medication before the scan (χ^2^ = 8.81, *p* < 0.05). Educational level demonstrated significant associations with several items, including whether the participants had sufficient information about MRI before the scan (χ^2^ = 41.23, *p* < 0.01), the use of anti-anxiety medication before the scan (χ^2^ = 17.33, *p* < 0.05) and the type of MRI machine used (χ^2^ = 21.23, *p* < 0.01). (Table 2). No statistically significant associations were observed for nationality.

Table 3 presents the results of differences in patient experience during examination according to demographic variables. Age showed significant associations across several aspects of patient experience, including overall experience (χ^2^ = 17.76, *p* < 0.01) and examination challenges (χ^2^ = 26.04, *p* < 0.01) (Table 3). Weight and educational level also demonstrated multiple significant associations across key aspects such as scan completion and communication with the technologist. Gender showed a more limited association, primarily related to overall experience, while nationality was associated with only one item related to sound preference during the examination.

Table 4 presents the results of differences in patient experience after examination according to demographic variables. Significant differences were observed for age (χ^2^ = 19.66, *p* < 0.05) and educational level (χ^2^ = 36.32, *p* < 0.01) in relation to the most uncomfortable feeling during the MRI examination (Table 4). Educational level was also significantly associated with both the presence of discomfort and its specific source (χ^2^ = 36.32, *p* < 0.01; Table 4).

**Figure 2:**
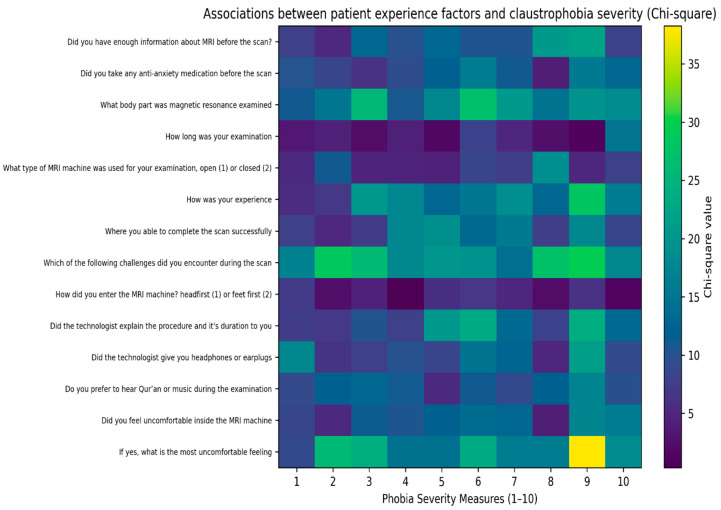
Heatmap of chi-square associations between patient experience variables and items of the Phobia Severity Scale. X-axis: Phobia Severity Measures (1–10); Y-axis: Patient Experience Variables [Before, During, and After MRI Examination].

Figure 2 presents associations between patient experience factors and individual items of the Phobia Severity Scale. Columns 1 to 10 represent specific claustrophobia severity items, ranging from emotional and cognitive symptoms (e.g., sudden fear, thoughts of injury) to behavioral patterns (e.g., avoidance, self-distraction) and physical manifestations (e.g., racing heart, tension). The colour intensity represents the magnitude of the Chi-square values, where higher Chi-square values indicate stronger associations between the variables, while lower values indicate weaker relationships. The coping strategy “Self-Distraction” showed several statistically significant associations with patient experience variables (Figure 2). This finding suggests that self-distraction may play a potentially important role in how some patients cope with MRI-related anxiety. It was significantly associated with receiving pre-scan information and a more positive overall experience during the examination (*p* < 0.001). This finding highlights the possible value of supportive preparation strategies that encourage coping techniques, such as self-distraction. The physical manifestations of anxiety, such as ‘Racing Heart, Trouble Breathing’ and ‘Tense Muscles, Restlessness’, were both significantly associated with the ability to complete the scan successfully (*p* < 0.05). This suggests that attention to somatic symptoms may help improve patient tolerance for the examination. Communication Mitigates Avoidance (Column 6): The avoidance behaviour ‘Avoided or did not approach/enter’ was significantly linked to the technologist’s explanation of the procedure. This may suggest a possible benefit of supportive preparation strategies that encourage coping techniques such as self-distraction. Overall, the associations shown in Figure 2 suggest that managing MRI-related claustrophobia may benefit from a tailored, multi-component approach that includes patient information, supportive staff communication and attention to patients with pronounced physiological symptoms of anxiety.

## 4. Discussion

The present study provides insight into the severity and patient-reported experience of claustrophobia and anxiety during MRI procedures in a Saudi cohort. Although most participants reported a generally positive experience, a notable proportion described anxiety-related symptoms; shortness of breath was a commonly reported discomfort. These results indicate that claustrophobia is still a clinically relevant issue in MRI practice and may negatively impact patients’ comfort and tolerance of the examination. In addition, the observed associations between the demographic factors and the anxiety-related responses indicate that an individual patient’s characteristics could play an important role in shaping the patient experience. In clinical practice, this highlights the importance of implementing structured patient preparation protocols and early screening for anxiety risk to improve patient tolerance and reduce the likelihood of incomplete MRI examinations.

Age demonstrated several statistically significant associations with claustrophobia-related responses, including emotional, cognitive, physiological, and behavioral symptoms. Differences were observed across age groups in reported fear, negative thoughts, anxiety-related symptoms, and coping strategies such as self-distraction. These findings could reflect the differences associated with age in controlling anxiety, inability to tolerate confined environments or the negative effect of previous medical experience. A possible mechanism underlying this observation is that older patients may have increased sensitivity to confined environments and reduced physiological adaptability to stress, which can amplify both emotional and somatic anxiety responses during MRI examinations. These findings are consistent with previous studies [24], which also reported that older patients are more likely to experience increased anxiety and require enhanced psychological preparation prior to MRI examinations. In contrast, nationality did not show statistical significance in phobia severity measures, which indicates that claustrophobia responses in this cohort are directly associated with individuals and procedural factors rather than cultural background. Clinically, these findings suggest that older patients may benefit from targeted psychological preparation, including reassurance, detailed explanation, and coping strategy guidance prior to MRI examination.

Male and female participants differed significantly on several emotional and cognitive severity measures, including sudden fear, anxiety and negative outcome expectations. This finding is consistent with Masalma et al. (2024) [15], who reported a higher prevalence of anxiety symptoms among females, supporting the role of gender-related psychological factors in anxiety perception. Psychological, social, and emotional expression and differential stress might contribute to these differences, which would confirm the need for individualised communication strategies to be considered when addressing gender differences during MRI procedures. In addition, weight showed significant associations with fear, avoidance and the need for coping support, which could be interpreted as physical restrictions imposed by closed MRI system designs. This difference may be explained by variations in stress perception, hormonal influences, and coping styles, which have been shown to affect anxiety responses in clinical settings. Obese patients could suffer from confined feelings, restricted environments and temperature discomfort; these factors could increase claustrophobic reactions [23]. Mechanistically, the reduced physical space within the scanner and increased body contact with the bore may heighten sensory awareness and discomfort, thereby triggering anxiety responses. These findings suggest that examination characteristics and patient position may influence patient comfort during examinations [25]. Interestingly, no statistically significant association was observed between claustrophobia severity and the type of MRI scanner (open vs. closed) or the entry position (headfirst vs. feetfirst). This may reflect the distribution of examination types in the sample or the influence of other factors, such as patient expectations, prior experiences and communication with MRI technologists. These findings indicate that MRI protocols may need to be adapted based on patient characteristics, such as providing additional support for patients with higher anxiety risk or physical discomfort to improve examination tolerance.

Moreover, educational level revealed a strong association with cognitive responses related to anxiety, particularly feelings of worry and nervousness. Patients with high levels of education could be more aware of medical procedures and the possible risks, which could conversely increase the predicted anxiety in the absence of reassurance. This finding aligns with previous research [26,27], which suggests that increased awareness of medical procedures may elevate anticipatory anxiety in the absence of adequate reassurance. This may be due to heightened cognitive anticipation, where increased knowledge leads to greater awareness of potential risks, thereby amplifying anxiety in uncertain situations. From a clinical perspective, this emphasizes that providing information alone is insufficient; the quality and clarity of communication must be tailored to the patient’s level of understanding to effectively reduce anxiety.

This finding is in agreement with previous studies [16,24], which demonstrated that adequate pre-scan information significantly reduces anxiety and improves patient compliance during MRI examinations. This effect may be mediated by reducing uncertainty and perceived threat, which are key drivers of anxiety in unfamiliar medical environments. These findings support the idea that the patient education concept is not only an informational process but also an effective intervention for reducing anxiety [28]. This is reinforced by the integration of structured patient education programs, including pre-scan counseling or multimedia tools, as a routine part of MRI workflow to reduce anxiety and improve patient experience. Conversely, the use of anti-anxiety medications is significantly associated with greater fear severity and avoidance indicators. However, given the cross-sectional nature of the study, the direction of this association cannot be determined. It is possible that patients with more severe claustrophobia symptoms were more likely to use anti-anxiety medication prior to MRI examinations, rather than medication use contributing to increased anxiety severity. This observation may reflect the tendency for pharmacological interventions to be used more frequently among patients with higher anxiety severity [29]. This may indicate that pharmacological interventions are often introduced after anxiety has already escalated, rather than targeting early psychological triggers of claustrophobia. This highlights the potential value of identifying patients at risk of anxiety and considering non-pharmacological coping strategies when appropriate.

Patient experience during the MRI examination was strongly influenced by the quality of interaction with MRI technologists. A clear explanation of the examination and its duration was significantly associated with reduced avoidance behaviour and improved emotional responses. This may be explained by the reduction in uncertainty and increased patient trust, which are known to mitigate anxiety responses in clinical procedures. This result highlights the important role of MRI technologists in shaping patient experience during MRI examinations [30]. This underscores the critical role of MRI technologists not only as technical operators but also as key contributors to patient-centered care, where effective communication can directly influence examination success. This observation is comparable to the findings described previously by Al-Shemmari et al. (2022), who reported that communication skills directly impact patient compliance and better tolerance of the MRI examination [21]. In addition, providing headphones and earplugs as well as auditory distraction, such as music or Qur’an preferences, was strongly associated with experience improvement and anxiety reduction [31]. These sensitive interventions represent simple, low-cost approaches that can be integrated easily into routine MRI practices to support patient comfort [32].

One of the most observed clinical outcomes of this study was the strong association between coping strategies and fear severity measures. Self-distraction demonstrated several statistically significant associations with multiple patient experience variables and dimensions of fear severity. It demonstrated a significant association with multiple variables of patient experience and various dimensions of fear severity. This is consistent with earlier studies [33], which have identified distraction techniques as effective non-pharmacological strategies for reducing anxiety during MRI procedures. In practice, incorporating simple coping strategies such as guided breathing, distraction techniques, or audio-based interventions into MRI preparation protocols may significantly improve patient tolerance and reduce scan interruptions. In addition, somatic manifestations of anxiety, including tachycardia, muscle tension and shortness of breath, showed a strong association with incompletion of the scan, which suggests that unmanaged physical symptoms may reduce patient tolerance of the examination [11]. Physiologically, these symptoms may reflect activation of the sympathetic nervous system, which can intensify the perception of distress and lead to premature termination of the scan.

After the examination, the feeling of discomfort was influenced by age, weight and educational level, especially in relation to the type and source of discomfort felt by the patient who underwent the MRI. This indicates that the physical and cognitive factors could continue even after scan completion, which confirms the need for comprehensive anxiety management over the course of the MRI examination rather than concentrating on only the scan phase [25]. Overall, these findings highlight that MRI-related claustrophobia can be effectively managed through a combination of patient-centered communication, tailored preparation, and supportive coping strategies integrated into routine clinical practice.

The findings of this study are consistent with previous research [24,34], which emphasizes the importance of patient education, effective communication, and coping strategies in improving MRI tolerance and reducing anxiety-related complications. This study contributes region-specific evidence on MRI-related claustrophobia and patient-reported experiences among patients in Saudi Arabia, where published data on this topic remain limited. Although this study offers valuable insight into patient anxiety about MRI procedures, several limitations should be acknowledged. The reliance on a convenience sample and the relatively small sample size (*N* = 200) restrict the generalizability of the findings to the broader Saudi population and may have reduced the statistical power to detect weaker associations between demographic variables and claustrophobia-related responses. In addition, the use of convenience sampling may have introduced selection bias, as individuals who agreed to participate may have differed in their anxiety perception and MRI experiences compared with those who declined participation. The significant demographic skew towards female participants and those with higher educational attainment may not accurately reflect the experiences of the entire patient cohort. This imbalance may have influenced the reported anxiety patterns, particularly in relation to gender- and education-related differences, and should be considered when interpreting these results. Furthermore, reliance on self-reported measures for anxiety and experience introduces the potential for recall and social desirability bias. In addition, the time elapsed since participants’ MRI examinations was not standardized or recorded, and therefore, the recall period may have varied considerably between participants. This variability could have influenced the accuracy and consistency of reported emotional responses and patient experiences, as participants with more distant MRI experiences may have underreported or inaccurately recalled anxiety-related symptoms compared with those who underwent MRI more recently. As a result, the reported levels of anxiety and coping behaviors may be either overestimated or underestimated, particularly for subjective measures such as fear perception and emotional responses. In addition, detailed MRI scanner specifications, such as magnetic field strength (e.g., 1.5 T vs. 3 T), were not collected. This limitation may have introduced unmeasured variability, as differences in scanner design, bore size, and acoustic noise could directly influence patient comfort and claustrophobia responses, and therefore should be considered when interpreting the findings. Future research should employ larger probability-based samples and incorporate objective physiological measures (e.g., heart rate variability) to corroborate self-reported distress. Additionally, the cross-sectional design of the study limits the ability to establish causal relationships between the identified factors and claustrophobia severity; therefore, the findings should be interpreted as associative rather than causal. Although the phobia severity scale was based on the DSM-5 Severity Measure for Specific Phobia—Adult and demonstrated excellent internal consistency in the current sample, formal psychometric validation of the Arabic-translated version was beyond the scope of the present study. In addition, the statistical analysis primarily relied on Chi-square tests without multivariable adjustment; therefore, potential confounding and collinearity between demographic variables could not be fully controlled. Accordingly, the findings should be interpreted as associative rather than indicative of independent predictors.

## 5. Conclusions

Claustrophobia remains a significant challenge in MRI clinical practice. The findings of this study suggest that patient-reported MRI experience may be improved through patient-centred strategies, particularly clear pre-scan education, effective communication with MRI technologists and supportive coping approaches. These measures may help reduce anxiety-related distress and improve the tolerance of MRI examinations.

## Figures and Tables

**Table 1:** Participant characteristics and selected questionnaire responses.

General Information		*N*	%
Age	from 18 to 29 years	103	51.5%
	from 30 to 40 years	32	16.0%
	more than 40 years	65	32.5%
Weight	less than 50 kg	31	15.5%
	from 50 to 70 kg	113	56.5%
	more than 70 kg	56	28.0%
Gender	male	37	18.5%
	female	163	81.5%
Educational level	Primary or Middle school	2	1.0%
	high school	33	16.5%
	Diploma	15	7.5%
	Bachelors	137	68.5%
	Master or PhD	13	6.5%
Nationality	Saudi	192	96.0%
	other	8	4.0%
Do you have caffeine consumption habits [coffee, energy drinks, tea]?	no	91	45.5%
	maybe	51	25.5%
	yes	58	29.0%
Do you suffer from shortness of breath in narrow places?	no	87	43.5%
	maybe	59	29.5%
	yes	54	27.0%
Have you been in situation that might have led to claustrophobia in your childhood?	no	159	79.5%
	maybe	20	10.0%
	yes	21	10.5%
What was the situation?	Stuck in the elevator	17	8.5%
	Confinement as punishment	22	11.0%
	drowning	7	3.5%
	Stuck in the bathroom	2	1.0%
	Stuck in the car	2	1.0%
	I do not remember	62	31.0%
	Nothing	80	40.0%
	other	8	4.0%
Did a relative or friend convey a bad experience with MRI to you?	no	115	57.5%
	maybe	17	8.5%
	yes	68	34.0%

**Table 2:** Differences in patient experience and MRI examination characteristics according to demographic variables.

	Chi-Square				
Items	Age	Weight	Gender	Education	Nationality
Did you have enough information about MRI before the scan?	9.05	5.98	2.98	41.23 **	1.03
Did you take any anti-anxiety medication before the scan?	11.72 *	8.67	8.81 *	17.33 *	0.467
Which body part was examined during the MRI scan?	9.85	14.74	9.09	18.74	0.834
How long was your examination?	0.904	4.70	0.021	2.36	0.373
What type of MRI machine was used for your examination, open [1] or closed [2]	5.36	6.95 *	0.008	21.23 **	3.419

* Significant at 0.05; ** Significant at 0.01.

**Table 3:** Differences in patients’ experiences during the examination according to demographic variables.

	Chi-Square				
Items	Age	Weight	Gender	Education	Nationality
How was your experience	17.76 **	4.79	9.97 *	18.68 *	0.989
Were you able to complete the scan successfully?	7.88	12.20 *	4.96	26.16 *	0.279
Which of the following challenges did you experience during the scan?	26.04 **	12.65	0.408	21.14	1.57
How did you enter the MRI machine: headfirst [1] or feet first [2]?	1.36	9.51 *	0.345	1.82	0.050
Did the technologist explain the procedure and its duration to you?	11.92 *	10.70 *	3.45	15.95 *	1.87
Did the technologist provide headphones or earplugs	9.02	6.50	4.04	16.44 *	1.56
Do you prefer to hear Qur’an or music during the examination?	19.83 **	13.37 *	2.25	19.52 *	6.64 *

* Significant at 0.05; ** Significant at 0.01.

**Table 4:** Differences in patients’ experiences after the examination according to demographic variables.

	Chi-Square				
Items	Age (χ^2^)	Weight (χ^2^)	Gender (χ^2^)	Education (χ^2^)	Nationality (χ^2^)
Did you feel uncomfortable inside the MRI machine?	7.14	5.89	5.83	19.67 *	0.745
If yes, what was the most uncomfortable feeling during the MRI examination?	19.66 *	16.12 *	3.83	36.32 *	2.05

* Significant at 0.05; Values represent χ^2^ statistics from Chi-square tests. *p* < 0.05.

## Data Availability

The data presented in this study are available from the corresponding author upon reasonable request, due to ethical and privacy restrictions.

## Supplementary Materials

The following supporting information can be downloaded at: https://www.mdpi.com/article/10.3390/tomography12060077/s1, Supplementary File S1, Questionnaire used for data collection in this study.
