# Supplementary material for: MRI-Related Claustrophobia: Patient-Reported Experience and Associated Factors in a Makkah Region Cohort
Source: Tomography. 2026 May 25;12(6):77. doi: 10.3390/tomography12060077 (PMC13306492; doi:10.3390/tomography12060077)
Supplement: Supplementary file 1 [file tomography-12-00077-s001.zip › tomography-4230061-supplementary.pdf]

استبيان عن الكلستروفوبيا او رهاب الاماكن الضيقة في جهاز الرنين المغناطيسي بهدف قياس وعي المجتمع بفحص الرنين المغناطيسي وتأثيره على الكلستروفوبيا /questionnaire about the claustrophobia in MRI in order to measure the community awareness of MRI and its effect on claustrophobia

السلام عليكم ورحمة الله وبركاته

نضع بين ايديكم استبانة بحثي يهدف قياس وعي المجتمع بفحص الرنين المغناطيسي و تأثيره على الكلستروفوبيا
This is questioner to measure community awareness in MRI and their effect on claustrophobia

جميع البيانات المشاركة في هذا الاستبانة يتم التعامل معها بسرية تامة ولن تستخدم الا لأغراض البحث العلمي / all the information in this questioner will be deal with strictly confidence and will only be used for research purposes

الفئة المستهدفة: كل من أجرى فحص الرنين المغناطيسي من المجتمع السعودي /the Saudi community who has been to the MRI examination

/thanks for your cooperative and support شاكرين لكم دعمكم وحسن تعاونكم

/for more information please contact at staldahery@uj.edu.sa للمعلومات

هل توافق على اجراء هذا الاستبيان؟
Do you agree to take this survey?

/yes نعم

/ no لا

معلومات عامة:

Demographic information:

/ Age العمر

/ less than 18 ١٨ اقل من√
/ 18_29٢٩√_١٨
/ 30_40 ٤٠√_٣٠
/ more than 40 ٤٠ اكبر من√

/Weight الوزن

/ less than 50 ٥٠ اقل من√
/ 50_70 ٧٠√_٥٠
/ more than 70 ٧٠ اكثر من√

/ Gender الجنس
/ male ذكر√
/ female أنثى√

/ the educational level المستوى التعليمي

/ Primary or Middle school ابتدائي او متوسط√
/ high school ثانوي√
/ Diploma دبلوم√
/ Bachelors بكالوريوس√
/ Master or PhD ماجستير او دكتوراة√

/ Nationality الجنسية

/ saudi سعودي√
غير ذلك...√

هل تعاني من ادمان الكافيين)قهوة، مشروبات طاقة، شاي (/ Are you a Coffein addicted (coffee, energy drinks , tea)l

/ yes نعم√
/ no لا√
/ maybe ربما√

هل تعاني من ضيق التنفس في الاماكن الضيقة
Do you suffer from shortness of breath in narrow places?

/ yes نعم√
/ no لا√

هل حصل لك موقف في طفولتك ادى الى الرهاب من الاماكن الضيقة
Have you been in situation that might have led to claustrophobia in your childhood?

/ yes نعم√
/ no لا√
/ maybe ربما√

/ What was the situation ? ما هو الموقف الذي ادى الى هذا الرهاب

/ Stuck in the elevator الحبس في المصعد√
/ Confinement as punishment العقاب بالحبس√
/ drowning الغرق√
/ Stuck in the bathroom الحبس في الحمام√
/ Stuck in the car الحبس في السيارة√
/ I do not remember لا اتذكر√

√غير ذلك...

هل قام أحد معارفك بنقل تجربة سيئة له من اشعة الرنين المغناطيسي؟
Has any of your relatives or friends conveyed a bad experience with MRI to you?

√نعم / yes

√لا / no

√ربما / maybe

هل سبق لك عمل فحص رنين مغناطيسي / Have you ever done an MRI scan ?

√نعم / yes

√لا / no

ملاحظة: إذا نعم ينقل المشارك في الاستبانة الي الأقسام التالية، إذا لا ينقل لصفحة Thank you for your participation.

تجربة المريض قبل الفحص:

هل كان لديك معلومات كافية عن الرنين المغناطيسي قبل عمل الفحص؟
Did you have enough information about MRI before the scan?

نعم / yes لا / no. ربما / maybe

هل اخذت اي ادوية مضادة للقلق قبل عمل الفحص
Did you take any anti-anxiety medication before the scan?

√نعم / yes

√لا / no

ما هي المنطقة التي تم عمل فحص الرنين المغناطيسي عليها؟
Which body part was examined by the magnetic resonance imaging?

√راس او رقبة / Head or Neck

√صدر / Chest

√اطراف علوية / Upper extremities

√بطن او حوض / abdomen or pelvis

√ اطراف سفلية/ Lower extremities

كم كانت مدة الفحص؟

How long was your examination?

١٥ √الى ٣٠ دقيقة / 15_30 minutes

√اكثر من ٣٠ دقيقة / more than 30 minutes

ما نوع الجهاز الذي استخدم في فحصك؟

الرنين المغناطيسي المفتوح)١ (او الرنين المغناطيسي المغلق)٢ (

What type of MRI machine was used for your examination, open (1) or closed (2)

خيار 1

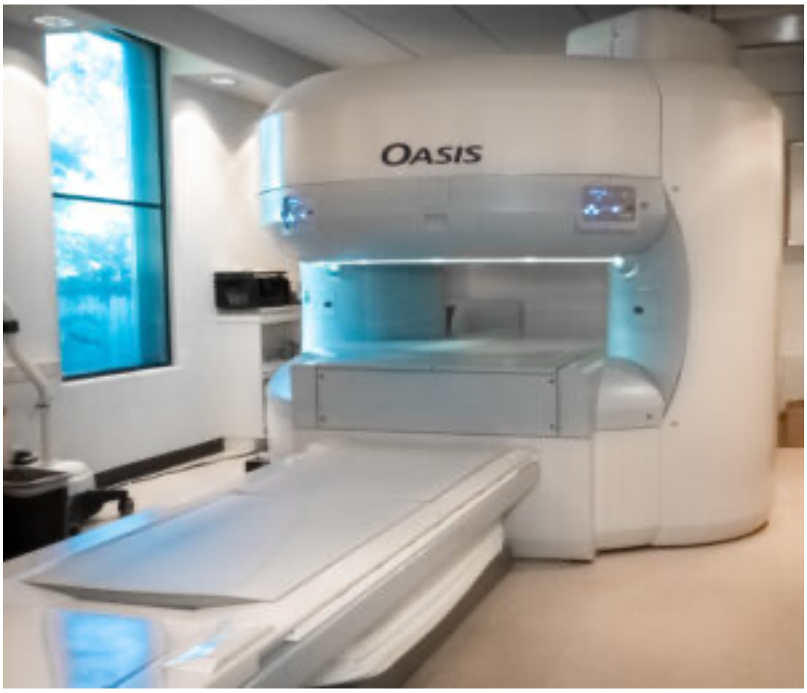

خيار 2

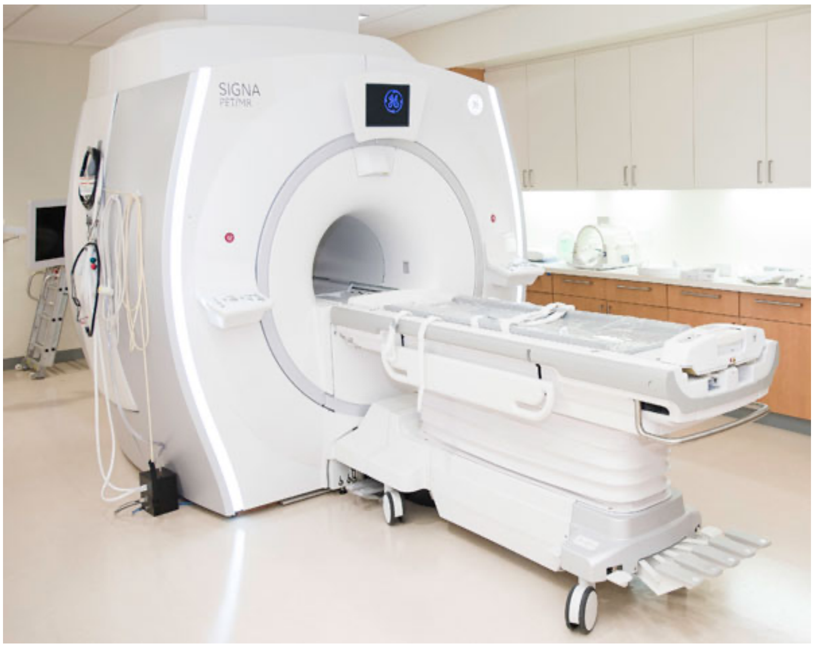

تجربة المريض اثناء الفحص:

Patient experience during the examination:

كيف كانت تجربتك ? How was your experience /

√ممتازة excellent /

√جيدة good /

√ سيئة/ bad

هل استطعت اكمال الفحص بنجاح؟

Were you able to complete the scan successfully?

√نعم yes /

√لا no /

ما هي التحديات التي واجهتك اثناء الفحص؟

Which of the following challenges did you encounter during the scan?

√ضيق في التنفس Shortness of breath /

√حركة لاارادية Involuntary movement /

√اغماء Fiant /

√تسارع ضربات القلب Fast Heartbeats /

√غير ذلك...

ما الاتجاه الذي تم وضعك به عند ادخالك جهاز اشعة الرنين المغناطيسي (ان توجه رأسك أولا نحو آلة الفحص) (١) او (أن توجه قدميك اولا نحو آلة الفحص) (٢)؟
How did you enter the MRI machine? head first (1) or feet first (2)?

خيار ١ / 1

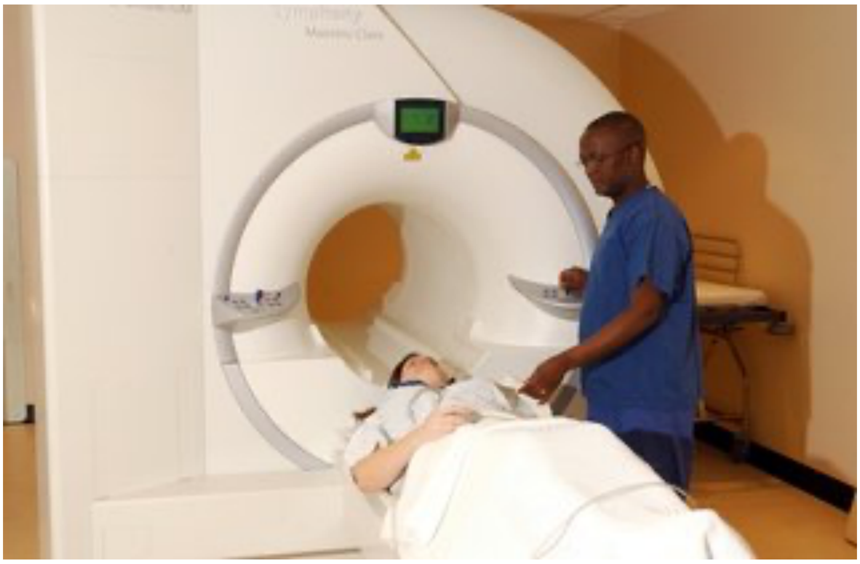

خيار ٢ 2 /

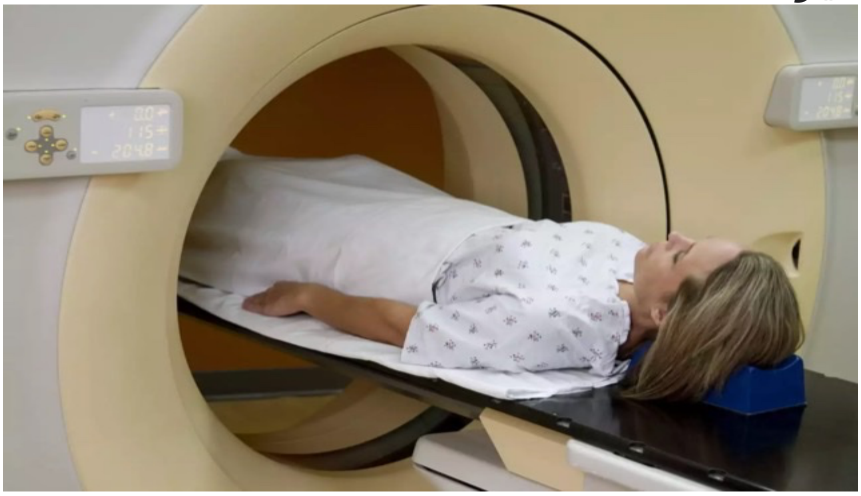

هل شرح لك اخصائي الاشعة عن الفحص ومدته؟
Did the technologist explain the procedure and its duration to you?

√نعم / yes

√لا / no
